# *Ficus deltoidea* promotes bone formation in streptozotocin-induced diabetic rats

**DOI:** 10.1080/13880209.2020.1865411

**Published:** 2021-01-05

**Authors:** Nurdiana Samsulrizal, Yong-Meng Goh, Hafandi Ahmad, Sulaiman Md Dom, Nur Syimal’ain Azmi, Noor Syaffinaz NoorMohamad Zin, Mahdi Ebrahimi

**Affiliations:** a Faculty of Applied Sciences, Universiti Teknologi MARA, Shah Alam, Malaysia; b Department of Veterinary Preclinical Sciences, Faculty of Veterinary Medicine, Universiti Putra Malaysia (UPM), Serdang, Malaysia; c Medical Imaging Department, Faculty of Health Sciences, Universiti Teknologi MARA, Shah Alam, Malaysia; d Department of Cell and Molecular Biology, Faculty of Life Sciences and Biotechnology, Shahid Beheshti University G.C, Evin, Tehran, Iran

**Keywords:** Diabetes, osteoporosis, bone histomorphometry, micro-CT, insulin, antioxidant, osteocalcin, BALP, DPD, fatty acids

## Abstract

**Context:**

Diabetes mellitus increases the risk of bone diseases including osteoporosis and osteoarthritis. We have previously demonstrated that *Ficus deltoidea* Jack (Moraceae) is capable of reducing hyperglycaemia. However, whether *F. deltoidea* could protect against diabetic osteoporosis remains to be determined.

**Objective:**

The study examines the effect of *F. deltoidea* on bone histomorphometric parameters, oxidative stress, and turnover markers in diabetic rats.

**Materials and methods:**

Streptozotocin (STZ)-induced diabetic Sprague-Dawley rats (*n* = 6 animals per group) received one of the following treatments via gavage for 8 weeks: saline (diabetic control), metformin (1000 mg/kg bwt), and methanol leaves extract of *F. deltoidea* (1000 mg/kg bwt). A group of healthy rats served as normal control. The femoral bones were excised and scanned *ex vivo* using micro-computed tomography (micro-CT) for histomorphometric analysis. The serum levels of insulin, oxidative stress, and bone turnover markers were determined by ELISA assays.

**Results:**

Treatment of diabetic rats with *F. deltoidea* could significantly increase bone mineral density (BMD) (from 526.98 ± 11.87 to 637.74 ± 3.90). Higher levels of insulin (2.41 ± 0.08 vs. 1.58 ± 0.16), osteocalcin (155.66 ± 4.11 vs. 14.35 ± 0.97), and total bone n-3 PUFA (2.34 ± 0.47 vs. 1.44 ± 0.18) in parallel with the presence of chondrocyte hypertrophy were also observed following *F. deltoidea* treatment compared to diabetic control.

**Conclusions:**

*F. deltoidea* could prevent diabetic osteoporosis by enhancing osteogenesis and inhibiting bone oxidative stress. These findings support the potential use of *F. deltoidea* for osteoporosis therapy in diabetes.

## Introduction

Diabetes mellitus is a metabolic disorder characterised by hyperglycaemia. It has been demonstrated that diabetes is associated with several lower extremities orthopaedic conditions and complications that affect the quality of life (Ay et al. 2020). Diabetes accelerates bone loss, osteopenia, and osteoporosis by promoting osteoclast function (Wongdee and Charoenphandhu 2011). Reports of delayed bone healing (Ajami et al. 2014; Marin et al. 2018), reduced growth plate thickness (Stalvey et al. 2017), and increased cortical porosity (Osima et al. 2017) support the incidence of compromised bone quality in diabetic subjects. Therefore, appropriate attention is warranted for elucidating mechanisms underlying bone microstructure changes associated with diabetes.

Bone formation and bone resorption are known as two counteracting processes at remodelling sites. A failure in the delicate balance between these two processes leads to osteoarthritis and osteoporosis (Logar et al. 2007; Feng and McDonald 2011). Clinical and preclinical studies agree that an increase in osteoclast and decrease in osteoblast activity compromises bone strength (Bloomgarden 2009). Several studies have suggested that STZ-induced diabetes is an excellent model for understanding the pathophysiological mechanisms of bone loss in diabetes (Ying et al. 2020). The selective toxicity of STZ was used to create animal models of diabetic osteoporosis in mice (Coe et al. 2013; Chen et al. 2018) and rats (Guo et al. 2019; Zheng et al. 2020). Intriguingly, STZ induces similar bone pathologies changes as seen in human T1DM (Motyl and McCabe 2009). It has been demonstrated that STZ is not only particularly toxic to the insulin-producing beta cells of the pancreas but also the skeletal muscles. STZ is transported into the cell by the glucose-transport protein GLUT2 that would lead to DNA damage. Skeletal muscle and beta cells have high levels of GLUT2 expression, thus tend to be more susceptible to STZ-induced bone loss. It is important to note that skeletal muscle is a potential mediator and determinant of bone quality (Kalaitzoglou et al. 2019). These observations sit well with other studies showing that STZ reduced bone formation (Peng et al. 2016) and increased the number of active osteoclasts (Zheng et al. 2017). Xie et al. (2018) reported that STZ decreases BMD, and induces trabecular bone loss due to oxidative stress and hyperglycaemia.

Micro-CT is a non-invasive procedure for three-dimensional BMD measurement in small animals (rats and mice) (Ashton et al. 2015). Preclinical studies have consistently demonstrated that micro-CT provides several advantages over classical histomorphometry. Ay et al. (2020) reported an increase in cellularity and lacunar density in STZ rats based on information obtained from high-resolution synchrotron micro-CT. However, several important features such as osteoblasts, osteoclasts, resorption lacunae, or osteoid seams on the bone surface cannot be visualised (Burghardt et al. 2011). It is reasonable to suggest that micro-CT analysis in combination with the traditional techniques can produce a reliable diagnosis of osteoporosis.

*Ficus deltoidea* Jack (Moraceae) is an evergreen shrub or a small tree that is easily found in Malaysia and widely distributed in Southeast Asian countries such as Thailand, Sumatra, Java, Kalimantan, Sulawesi, and Moluccas. The decoction of *F. deltoidea* has traditionally been used in postpartum care specifically to improve uterine strength, regain energy, and prevent postpartum bleeding (Sulaiman et al. 2008; Salleh and Ahmad 2013). It is also served as a health tonic or taken as herbal tea to relieve headache, fever, and toothache (Bunawan et al. 2014). Acute toxicity studies showed that the LD_50_ of aqueous and ethanolic leaf extracts of *F. deltoidea* was greater than 5000 mg/kg body weight (Farsi et al. 2013) and 2000 mg/kg body weight (Nugroho et al. 2020), respectively. These observations suggest that *F. deltoidea* extract can be considered safe and non-toxic for therapeutic use. Our previous study showed that *F. deltoidea* is not only capable of reducing blood glucose but also improving tissue function, structure, and behavioural performance of diabetic rats (Nurdiana et al. 2018). In addition, treatment with *F. deltoidea* increased the percentage of circulating amylin and n-3 polyunsaturated fatty acids, in particular, docosahexaenoic acid (DHA) (Nurdiana et al. 2017). Amylin and DHA are known to have antidiabetic (Adeghate and Kalász 2011; Li et al. 2014) and bone protective effects (Horcajada-Molteni et al. 2001).

Although *F. deltoidea* has indeed shown potent anti-osteoporotic effects in the osteoporosis model (Che Ahmad Tantowi et al. 2018), its protective effect on diabetic osteoporosis remains unclear. This prompted us to determine the effects of *F. deltoidea* treatment on bone histomorphometric parameters, oxidative stress, and turnover markers in STZ-treated rats.

## Materials and methods

### Plant material and extract preparation

The leaves of *F. deltoidea* var. *deltoidea* were purchased from Moro Seri Utama Enterprise, Batu Pahat, Johor, Malaysia in September 2016. The plant material was identified and authenticated by Mr. Sani Miran, a specialised taxonomist. A voucher specimen (UKMB-40,315) was deposited in the Herbarium Unit, Universiti Kebangsaan Malaysia for further reference. The leaves were washed thoroughly, oven-dried at 37 ± 5 °C, ground to a fine powder in an electric grinder, and weighed. The powdered leaves (100 g) were soaked in 1 L absolute methanol for three days at room temperature. Liquid extracts were concentrated using a rotary vacuum evaporator (R-215, Buchi, Switzerland) under reduced pressure. The extracts were kept in tightly closed glass containers and stored at −20 °C until further use.

### Animals

The study was conducted with 24 male Sprague-Dawley rats, weighing 100–120 g, purchased from Chennur Suppliers, Malaysia. The animals were maintained in a temperature-controlled room (22 ± 1 °C and a 12 h light/dark cycle) and fed with standard rat chow (Gold Coin Holdings, Kuala Lumpur, Malaysia) and free access to water. The experiment protocols including diabetes induction and sacrifice operation were approved by the Universiti Putra Malaysia, Animal Care and Use Committee (UPM/IACUC/AUP-R090/2014).

### Induction of diabetes

Diabetes-like hyperglycaemia in rats was induced chemically by a single intraperitoneal injection of 60 mg/kg STZ dissolved in 10 mM citrate buffer (pH 4.5). To prevent the drug-induced hypoglycaemic shock, the rats were given 5% glucose water for 2 days following STZ injection (Gurukar et al. 2013). Animals with fasting blood glucose levels higher than 11 mmol/L after a week of injection were considered diabetic (Dong et al. 2014). The rats in the normal control group were injected with the same volume of isotonic NaCl.

### Experimental design

A total of 24 male rats were divided into four groups (*n* = 6). The assigned groups were as follows: normal control rats received saline (NC), diabetic control rats received saline (DC), Diabetic rats treated with 1000 mg/kg b.w. of metformin (DMET) and diabetic rats treated with 1000 mg/kg b.w. of *F. deltoidea* (DFD). Treatments were given via oral gavage once daily for a 56-day duration. At the end of the experiment, all animals fasted overnight and blood glucose levels were measured. Animals were then anaesthetised with ketamine (80 mg/kg) and xylazine (8 mg/kg), followed by terminal exsanguination. The femur and tibia were separated by cutting at the stifle joint. Blood samples (10–15 mL) were collected via cardiac puncture from the rats into a plain red-top tube containing no anticoagulants (BD Vacutainer^®^, USA). The blood samples were then centrifuged at 4000  *g* for 15  min, and serum was stored in aliquots at −80 °C.

### *Ex vivo* micro-computed tomography (μCT) analysis of bone

The bone tissue analyses were performed using SkyScan 1176 micro-CT scanner (Bruker, Kontich, Belgium) equipped with a 1 mm aluminium filter at a voltage of 41 kV and a current of 232 μA as described by Azmi et al. (2019). The rotation step size used for acquiring images was 0.6 degrees. The transverse 2 D cross-sectional images were reconstructed using NRecon software (Bruker-MicroCT). The images were then analysed for BMD, trabecular parameters (number, TbN; separation, TbSp; thickness, TbTh), and BV/TV (Bruker provides the CTAn software, ScancoMedical the IPL software).

To calculate the BMD (in mg/cm^3^) of each femoral diaphysis, a calibration curve was obtained by phantom experiments. This procedure allows the conversion of Hounsfield units (HU) into BMD values. The phantom was made up of a plastic base material, polyethylene; containing five cylindrical holes with 190 ± 0.5 mm diameter. Four cavities were filled with 50, 150 and 500 mg/cm^3^ solutions of K_2_HPO_4_ in distilled water, as known reference bone substitutes, and the fifth is a fat equivalent that was filled with 60% ethanol.

### Histological assessment

After micro-CT analysis, the samples were decalcified in 0.5 mmol/L EDTA phosphate-buffered saline (pH 7.4) at 4 °C for 2 months (Huang et al. 2014). The EDTA solution was changed every week. Followed by the dehydration and embedding in paraffin, the bone specimens were sectioned at 4 µm thickness and stained with haematoxylin and eosin. All slides were examined using light microscopy (Motic BA410, Wetzlar, Germany) equipped with a digital camera (Moticam Pro 285 A, Wetzlar, Germany), under a magnification of ×200 and ×400.

### Measurements of bone oxidative stress and antioxidant activities

The pieces of femur bone were ground using mortar and pestle. Bone tissues were homogenised in 10% (w/v) homogenising buffer (50 mM Tris-HCl, 1.15% KCl pH 7.4) using a Teflon pestle (Glass-Col, USA). The homogenates were centrifuged at 9000 *g* in a refrigerated centrifuge (4 °C) for 10 min to remove nuclei and debris. The obtained supernatant was assayed using commercial kits: TBARS assay kit for monitoring lipid peroxidation, glutathione peroxidase (GPx) assay kit for GPX activity, and superoxide dismutase (SOD) assay kit for SOD activity (Cayman Chemical, USA). Protein concentration was estimated by the method of Lowry et al. (1951), using bovine serum albumin as the standard.

### Marker of bone formation and bone resorption

All markers of bone formation and bone resorption were measured in serum. The osteocalcin level was determined by using Rat-Mid Osteocalcin ELISA kit (IDS, UK) whereas the rat BALP ELISA kit (Qayee, Shanghai) was used to examine the levels of BALP. To assess bone resorption DPD was measured by using Rat deoxypyridinoline (DPD) ELISA Kit (Qayee, Shanghai). All samples were run in triplicate and the optical density was read at 450 nm with a microplate reader (Epoch Microplate Spectrophotometer, BioTek, USA) as described by Abdul-Majeed et al. (2012).

### Analysis of bone fatty acid composition

The total fatty acids were extracted from bone, identified and quantified by gas chromatography method as described by Nurdiana et al. (2017). The fatty acid proportions are expressed as the percentage of total identified fatty acids.

### Statistical analysis

All data were tested using one-way ANOVA. Duncan’s multiple comparison test was employed to elucidate significant means. Results were presented as the mean ± 1 SD. All analyses were performed at 95% confidence level.

## Results

### Fasting blood glucose and serum insulin

The DC rats showed high fasting blood glucose and low insulin levels compared to the NC group (Table 1). Treatment with *F. deltoidea* significantly reduced the level of fasting blood glucose and significantly increased the levels of serum insulin in the diabetic rats.

**Table 1.** Effects of *F. deltoidea* on fasting blood glucose level and serum insulin in STZ induced diabetic rats (data represent mean ± 1SD).

Groups	Fasting blood glucose (mmol/L)		% Changes	Serum insulin (μIU/mL)
	Before	After		
NC	4.80 ± 0.30^a^	4.93 ± 0.21^a^	+2.71	4.16 ± 3.03^c^
DC	20.00 ± 3.24^b^	30.13 ± 2.63^b^	+50.65	1.58 ± 0.16^a^
DMET	29.30 ± 3.70^c^	19.83 ± 3.75^c^	−32.32	1.78 ± 0.34^a^
DFD	27.87 ± 6.03^c^	17.27 ± 4.97^c^	−38.03	2.41 ± 0.08^b^

Values with different superscripts down the column indicate significant difference at *p* < 0.05.

### Trabecular morphometric parameters

As depicted in Table 2, the DC rats showed a significant decrease in BMD, TbN, and BV/TV but TbSp was significantly increased than those of NC rats. In contrast, the TbN and BV/TV values increased following *F. deltoidea* treatment. It was also noticed that TbSp returned to near-normal levels and BMD increased significantly in response to *F. deltoidea* treatment.

**Table 2.** Histomorphometric results obtained on 2 D histological sections (data represent mean ± 1SD).

Groups	BMD (mg/cm^3^)	TbN (1/mm)	TbSp (mm)	TbTh (mm)	BV/TV (%)
NC	748.04 ± 12.11^d^	2.28 ± 0.07^c^	0.28 ± 0.01^a^	0.25 ± 0.01	56.66 ± 0.30^c^
DC	526.98 ± 11.87^a^	1.13 ± 0.01^a^	1.12 ± 0.00^c^	0.25 ± 0.01	28.33 ± 0.44^a^
DMET	606.52 ± 6.17^b^	1.66 ± 0.21^b^	0.79 ± 0.27^b^	0.18 ± 0.04	29.39 ± 4.34^a^
DFD	637.74 ± 3.90^c^	1.49 ± 0.29^ab^	0.53 ± 0.07^ab^	0.26 ± 0.05	38.68 ± 0.70^b^

Different superscripts ^a,b,c,d^ in a column differed significantly at *p* < 0.05.

### Micro-computed tomography images of bone

**Figure 1.**
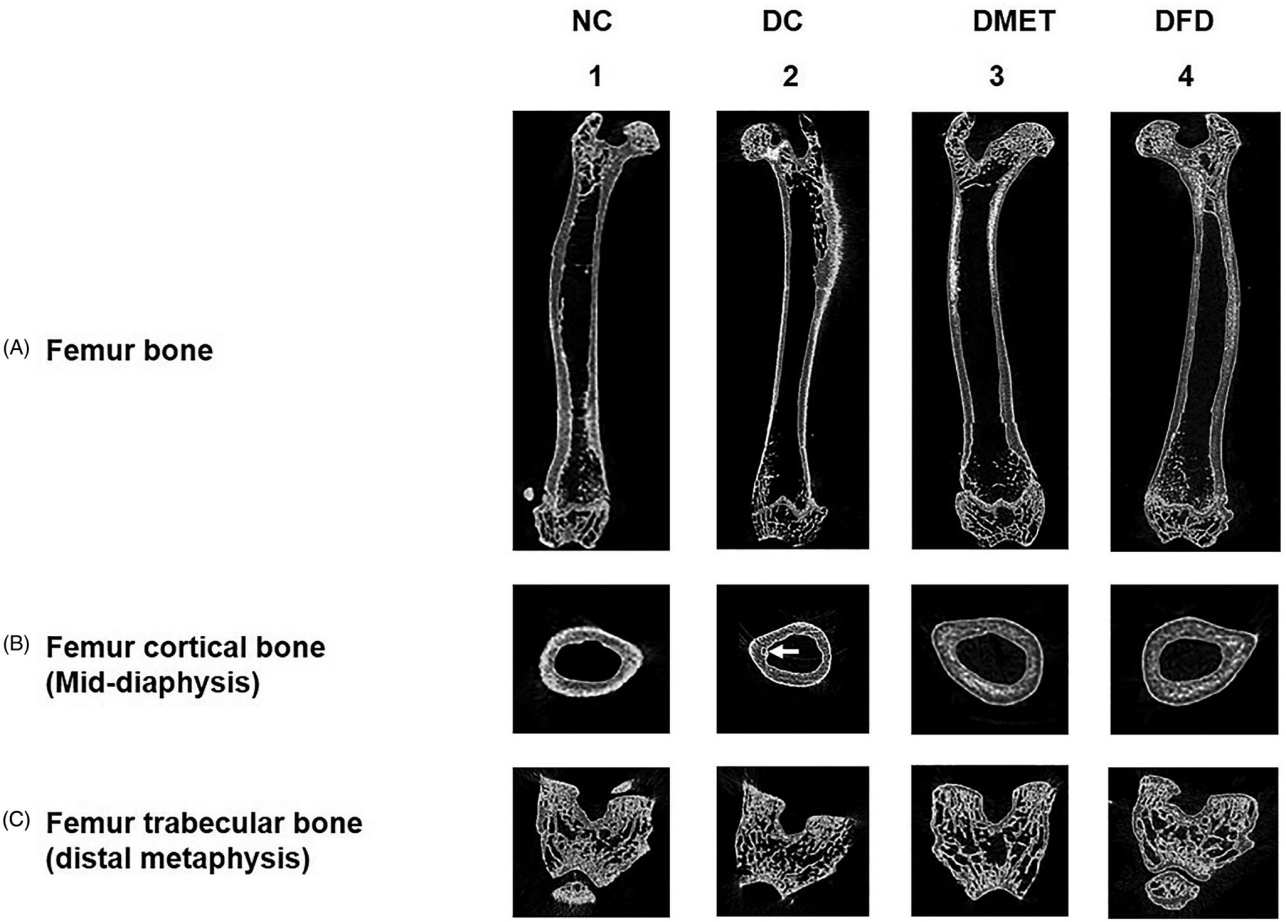
Micro-CT images of rat femurs in 2 D. (1) The NC rats had a thick layer of cortical bone surrounding dense bone trabeculae in the proximal and distal femur. (2) The DC rats display cortical osteopenia in the femoral diaphysis [indicated by white arrow] and trabecular bone loss in the distal femur. (3–4) Cortical bone thickness and density of bone trabeculae increased in the DMET and DFD rats. Images are representative of three animals per experimental group.

Micro-CT scanning analyses of the femoral bone showed that the DC rats exhibited cortical thinning (Figure 1(B2)) and trabecular bone loss (Figure 1(C2)). The cortical bone thickness increased markedly in the DMET (Figure 1(B3)) and DFD rats (Figure 1(B4)). Nevertheless, a remarkable increase of bone trabecular was observed only in the distal metaphysis of the DFD group (Figure 1(C4)).

**Figure 2.**
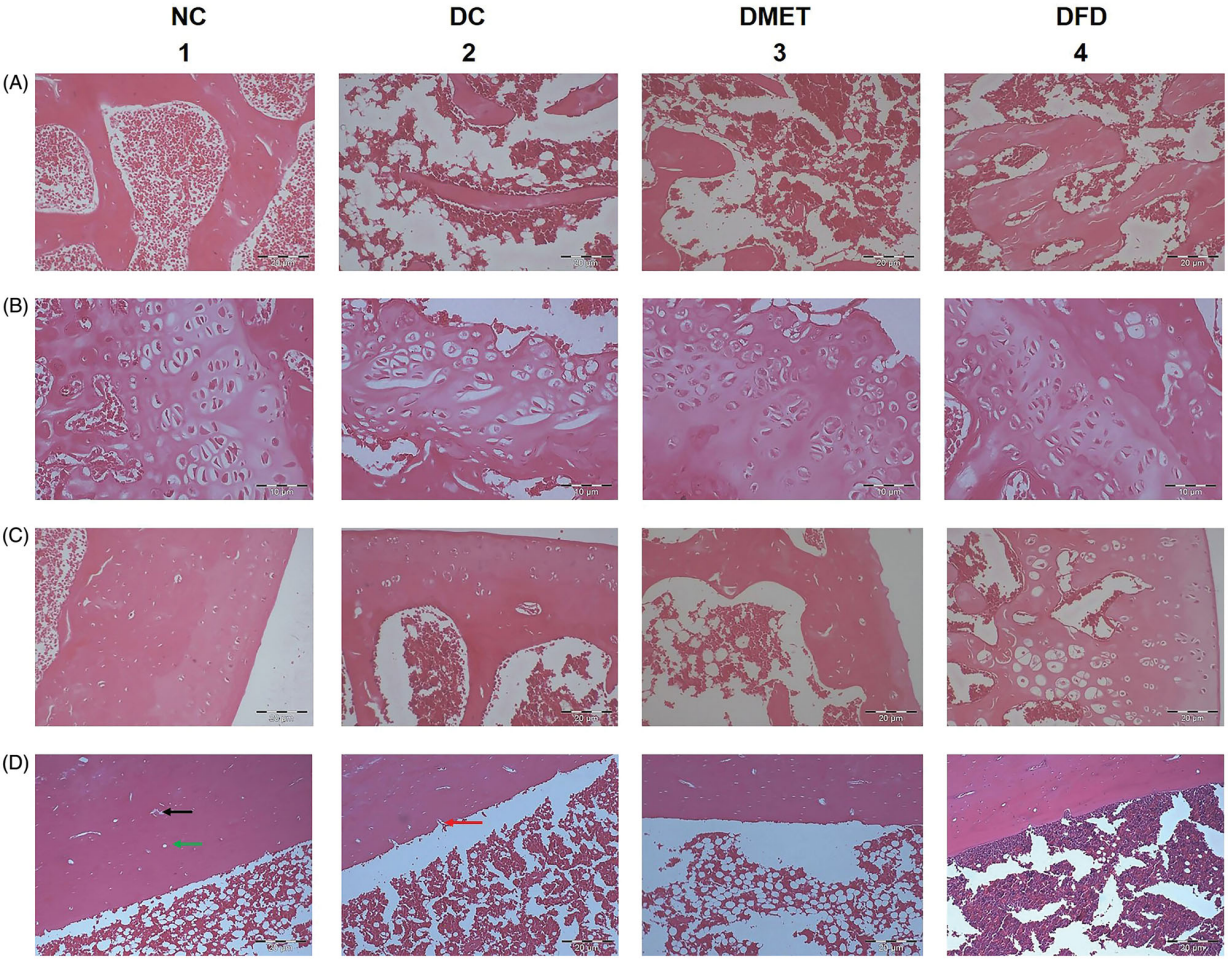
Light photomicrographs of sagittal sections of rat femur from different experimental groups. Normal control rats showing (A1) branching and anatomising thick trabeculae (T) separated by bone marrow (BM) spaces with some fatty tissue, (B1) well-developed growth plate, (C1) normal healthy articular cartilage, and (D1) cortical bone of femur diaphysis with haversian canals [indicated by green arrow] and osteocytes in their lacunae [indicated by black arrow]. Diabetic rats revealed (A2) thinning of the bone trabeculae with widening of the bone marrow spaces, (B2) disruption of the growth plate, (C2) reduction in articular cartilage quality at the femoral condyle, (D2) Multiple eroded [indicated by red arrow] areas at the endosteal surface of the cortical bone were obtained. Diabetic rats treated with metformin showing (A3) sparse and thinning trabeculae, with loss of connectivity and presence of abundant adiposity cells, containing a noticeable increased fatty tissue, (B3) wider distal femur growth plate, (C3) erosion of articular cartilage, and (D3) increase cortical porosity. Diabetic rats treated with F. deltoidea displaying (A4) larger areas covered with trabeculae and increased bone matrix density, (B4) epiphyseal plate arranged in layered array, (C4) thicker calcified cartilage component, and (D4) less cortical erosion. (A) Distal metaphysis trabecular (magnification 200×); (B) Distal epiphylseal plate (magnification 400×); (C) articular cartilage (magnification 200×) and (D) Cortical bone of femur diaphysis (magnification 200×).

### Bone histology

The histological section of normal rat femur showed a network of bone trabeculae at the distal femoral metaphysis which separated by bone marrow spaces (Figure 2(A1)). It was also found that osteocytes were surrounded by their lacunae in the bone matrix. As depicted in Figure 2(C1), a normal healthy articular cartilage was observed on the distal femur of NC rats in which the calcified cartilage layer is flanked by an undulating tidemark. Conversely, the DC rats displayed a sparse and thinner trabecular structure of the cancellous bone (Figure 2(A2)) along with a paucity of cells in the proliferative zone (Figure 2(B2)). The thickness of the cortical layer and articular cartilage was phenotypically decreased in the DC rats. Strikingly, it was observed that the bone trabeculae were more orderly arranged and bone matrix density increased (Figure 2(B4)) in the DFD rats. Figure 2(D4) also showed that less cortical erosion was found following *F. deltoidea* treatment. However, the DFD rats were associated with thicker calcified cartilage with chondrocyte hypertrophy.

### Oxidative stress marker and antioxidant enzymes in bone

Table 3 summarises the results for the effect of *F. deltoidea* on bone lipid peroxidation and the activities of antioxidant enzymes. It was found that the DC rats showed a significant increase in the levels of MDA with no significant difference in GPx and SOD activities as compared to the NC rats. A similar observation is seen in the DFD rats.

**Table 3.** Oxidative stress marker and antioxidant enzymes of various experimental groups (data represent mean ± 1SD).

Groups	Oxidative stress marker	Antioxidant enzymes	
	TBARS (nmol MDA/mg protein)	GPx (U/mg protein)	SOD (mU/mg protein)
NC	29.73 ± 0.50^a^	43.65 ± 0.78^ab^	0.51 ± 0.01
DC	60.74 ± 0.66^b^	44.40 ± 0.80^bc^	0.31 ± 0.04
DMET	73.51 ± 8.20^c^	42.06 ± 0.98^b^	0.34 ± 0.04
DFD	75.79 ± 0.14^c^	45.41 ± 0.46^bc^	0.58 ± 0.18

Different superscripts ^a,b,c^ in a column differed significantly at *p* < 0.05.

### Bone turnover markers

The injection of STZ resulted in a sharp drop in serum osteocalcin and ALP while serum DPD was significantly higher than the NC group (Table 4). Although there were no significant differences in the BALP value of all treated groups, the levels of serum osteocalcin increased while DPD decreased following *F. deltoidea* treatment.

**Table 4.** Changes in serum osteocalcin, BALP and DPD of various experimental groups (data represent mean ± 1SD).

Groups	Bone formation markers		Bone resorption marker
	Osteocalcin (ng/ml)	BALP (ng/ml)	DPD (ng/ml)
NC	137.78 ± 6.92^c^	101.49 ± 7.59^b^	157.08 ± 5.33^b^
DC	14.35 ± 0.97^a^	67.06 ± 4.70^a^	167.10 ± 0.21^c^
DMET	57.42 ± 8.24^b^	81.38 ± 0.45^a^	152.16 ± 4.08^ab^
DFD	155.66 ± 4.11^d^	77.30 ± 8.31^a^	145.53 ± 0.41^a^

Different superscripts ^a,b,c,d^ in a column differed significantly at *p* < 0.05.

### Bone fatty acid changes

The recorded data in Table 5 shows the total *n* − 3 PUFA was significantly decreased while the ratio of *n* − 6 to *n* − 3 PUFA was significantly increased in the femur of DC rats. Similar observations were noticed in the DMET group. Remarkably, the total bone *n* − 3 PUFA increased and the *n* − 6 to *n* − 3 ratio decreased in the DFD group.

**Table 5.** Fatty acid composition (percentage of total identified fatty acids) of the bone of the experimental groups (data represent mean ± SD).

Fatty acids composition (%)	Groups			
	NC	DC	DMET	DFD
Myristic acid (C14:0)	1.50 ± 0.27^c^	0.40 ± 0.05^a^	1.10 ± 0.08^bc^	0.61 ± 0.03^ab^
Palmitic acid (C16:0)	24.38 ± 4.58	24.88 ± 3.47	28.17 ± 2.88	23.34 ± 12.78
Stearic acid (C18:0)	7.07 ± 1.01^a^	8.88 ± 0.52^b^	7.13 ± 0.80^a^	9.43 ± 1.72^b^
Palmitoleic acid (C16:1)	2.52 ± 0.51	1.57 ± 0.38	1.68 ± 0.41	1.76 ± 0.64
Oleic acid (C18:1n9)	20.62 ± 7.99	25.02 ± 4.73	27.61 ± 3.12	23.66 ± 3.00
Linoleic acid (C18:2n6)	3.04 ± 1.37	3.27 ± 0.25	4.19 ± 0.42	2.69 ± 0.39
Arachidonic acid (C20:4n6)	1.00 ± 0.08	1.68 ± 0.04	1.04 ± 0.06	2.12 ± 0.14
α- Linolenic acid (C18:3n3)	1.55 ± 0.45	1.16 ± 0.29	1.24 ± 0.52	1.36 ± 0.96
Eicosapentaenoic acid (C20:5n3)	0.62 ± 0.15	0.14 ± 0.05	0.27 ± 0.05	0.68 ± 0.15
Docosapentaenoic acid (C22:5n3)	0.38 ± 0.02	0.57 ± 0.04	0.50 ± 0.24	0.38 ± 0.15
Docosahexaenoic acid (C22:6n3)	0.65 ± 0.16	0.14 ± 0.01	0.28 ± 0.03	0.31 ± 0.03
total SFA	32.94 ± 5.86	34.16 ± 4.32	36.39 ± 3.53	33.38 ± 13.01
total MUFA	23.13 ± 7.51	26.59 ± 4.72	29.29 ± 2.76	25.43 ± 3.44
total n-6 PUFA	4.04 ± 1.96	4.96 ± 0.79	5.23 ± 0.98	4.81 ± 1.39
total n-3 PUFA	2.81 ± 0.60^c^	1.44 ± 0.18^a^	1.78 ± 0.24^ab^	2.34 ± 0.47^bc^
n-6 : n-3	1.44 ± 0.69^a^	3.44 ± 0.23^d^	2.94 ± 0.34 ^cd^	2.03 ± 0.20^ab^

## Discussion

It is well known that bone strength is determined by BMD and the spatial structure of trabecular bone (Wakabayashi et al. 2004). The data from the micro-CT scan showed that the DC rats had a significant decrease in BMD, TbN, and BV/TV, as well as an increase in TbSp value. Similar findings were reported by Zhou et al. (2015), suggesting that STZ-induced diabetic rats are associated with trabecular loss and cortical osteopenia (Rao Sirasanagandla et al. 2014). One particularly interesting finding was that TbN and BV/TV values increased following *F. deltoidea* treatment (Table 2). A greater cortical thickness with a subsequent increase in BMD was also observed in the DFD rats. These findings suggest the possibility of *F. deltoidea* treatment for delaying the progression of osteoporosis in diabetes. Consistent with our findings, it has been demonstrated that plants with antiosteoporotic activity significantly increased the thickness of cortical and trabecular bone as well as improved BMD in the rat (Kumar et al. 2010; Suthon et al. 2016).

Articular cartilage is known to lubricate the ends of bones, thus, changes in articular cartilage can ultimately result in osteoarthritis. We further observed, via H&E staining, that injection of STZ leads to a decrease in the thickness of the femoral articular cartilage, lower chondrocyte numbers, and increased tidemark roughness. Some reports explained the cartilage becomes hypocellular in the late stages of osteoarthritis (Hwang and Kim 2015). In line with our results, Xu et al. (2014) showed the roughness of tidemark increased with loss of chondrocyte and irregular arrangement in the osteoarthritis mice. These findings together indicate the progression of osteoarthritis-like disorder in diabetic rats. Indeed, the osteoarthritis-like consequences have been reported in T1DM and T2DM rats (Onur et al. 2014; King and Rosenthal 2015). Activation of oxidative stress is thought to be partially responsible for these changes. However, this hypothesis warrants further investigation. Of interest, thicker calcified cartilage with chondrocyte hypertrophy was observed in the DFD rats. Permuy et al. (2015) showed that thickened articular cartilage and chondrocyte hypertrophy is a distinctive feature of the early stages of osteoarthritis in animal models. Several studies reported that inhibition of chondrocyte hypertrophy represents a therapeutic target to slow down further osteoarthritis progression (Yahara et al. 2016). Based on these findings, we hypothesised that *F. deltoidea* treatment is effective in delaying the pathogenetic progression of osteoarthritis in diabetic rats.

Both clinical and preclinical studies pointed out that the pathogenesis of osteopenia, osteoporosis, and osteoarthritis are influenced by oxidative stress (Guo et al. 2013; Lepetsos and Papavassiliou 2016). Therefore, it is worth further deciphering the relation between oxidative stress and bone quality. Herein, we showed that the DC rats were associated with increased levels of oxidative damage markers. A significant increase in MDA level was also observed in all treated animals, and thereby strengthened susceptibility to STZ-induced bone complications in animal studies (Yee et al. 2016). It has been shown that oxidative stress along with hyperglycaemia can disrupt bone metabolism and architecture by altering the function of osteoclast and osteoblast (Lee et al. 2015). Furthermore, hypertrophic chondrocyte-like phenotype has been postulated to be a consequence of oxidative stress (Kishimoto et al. 2010). This was relevant to our observations that the DFD rats, which show such a high number of chondrocyte hypertrophy, had among the highest MDA levels. What is more, high plasma MDA concentrations are related to the early stages of osteoarthritis (Martins et al. 2015), thus strengthening the hypothesis that *F. deltoidea* can delay the progression of osteoarthritis.

As oxidative stress can destabilise the balance between osteoblast and osteoclast activities, it provides a strong rationale for the measurement of bone turnover markers (Starup-Linde 2013). The present study demonstrated that serum DPD levels increased while serum osteocalcin and BALP activities decreased in the DC rats. This finding is in agreement with Zhukouskaya et al. (2015), who showed that suppression of bone turnover is the main characteristic of T1DM-associated bone disorder. These data concur with previous reports of increased serum DPD in rats with osteoarthritis (Lee et al. 2016) and osteopenia (Abuohashish et al. 2013).

Another important finding from the present study is that the levels of serum osteocalcin increased while DPD decreased following *F. deltoidea* treatment (Table 4). Similar observations have been made on several plants with osteoprotective effects (Song et al. 2016). Although osteocalcin is a specific osteoblast marker and highly correlates with histological changes (Gundberg et al. 2012), the level of serum OC tended to fluctuate with food intake (Starup-Linde 2013). Earlier work indicated that osteocalcin does not appear to be as sensitive marker as BALP (Kaddam et al. 1994). Indeed, the activity of BALP remains low in the DFD rats, indicating a continuing adverse effect on mineral metabolism. BALP is the bone-specific isoform of alkaline phosphatase, synthesised by the osteoblasts for bone remodelling process, but more specifically reflects mineral metabolism (Cheung et al. 2013). Of interest, the ratio between osteocalcin to DPD was nearly equal to those of the NC groups, suggesting an equilibrium between bone formation and bone resorption almost achieved with *F. deltoidea* treatment.

The present studies have also indicated that there is a close relationship between bone health and fatty acids profile. The DC and DMET rats with histological and biochemical evidence of bone loss and osteoarthritis-like disorder showed a significant decrease in total bone *n* − 3 PUFA and an increase in *n* − 6: *n* − 3 ratio (Table 5). These changes, however, have greatly improved following *F. deltoidea* treatment. Recent work by Longo and Ward (2016) has shown that a high intake of *n* − 3 PUFA was possible to increase BMD and reduce the risk of fragility fracture. Previous works also demonstrate that *n* − 3 PUFA supplementation may have a protective effect on bone metabolism by decreasing bone resorption markers (Griel et al. 2007). Indeed, the BMD was much higher (Table 2) and the DPD was much lower (Table 4) in the DFD rats compared to other animals treated with STZ. This finding lends additional evidence to further support the potential of *F. deltoidea* treatment against bone loss in STZ-treated rats.

## Conclusion

Our data confirm that *F. deltoidea* has the potential to prevent bone loss in STZ-treated rats. Treatment with *F. deltoidea* significantly reduced the levels of fasting blood glucose, DPD activity, and increased insulin secretion, osteoblast activity, BMD, TbN, and BV/TV.
